# Letter to the Editor regarding article “Electrocardiographic markers of increased risk of sudden cardiac death in patients with COVID‐19 pneumonia”

**DOI:** 10.1111/anec.12868

**Published:** 2021-07-21

**Authors:** Fenglin Jiang

**Affiliations:** ^1^ Department of Ultrasound Medicine Yanbian University Hospital Yanji China

## CONFLICTS OF INTEREST

The authors have no potential conflicts of interest to disclose.

## AUTHOR CONTRIBUTIONS

Conceptualization,Writing ‐ original draft,Writing ‐ review & editing: Fenglin Jiang.

Dear Editor,

We have read with great interest the article “Electrocardiographic markers of increased risk of sudden cardiac death in patients with COVID‐19 pneumonia” by Alareedh et al., (2021).

The difference between respiratory system virus, circulatory system virus, pulmonary hemorrhagic fever virus, and enterovirus is not very important. Is there still less virus in *E. coli* culture? ACE2 mediates the destruction of endothelial cells, and thrombosis may be the mechanism. Where is prethrombotic state and hypercoagulable state? It is just an academic discussion of assumptions. The ECG of pulmonary embolism is the camouflager of the heart world. It has various manifestations. Autopsy seems to have proved everything and myocardial infarction.

Acute cardiac injury incidence in COVID‐19 is about 13 times higher in the intensive care unit /severely ill than in less critical patients. The cytokine release with complement and iNO dysregulation are established mechanisms potentially leading to sepsis‐related cardiomyopathy, making sepsis per se one of the potential mechanism leading to acute cardiac injury in COVID‐19 patients. Moreover, the hyper‐inflammation with endothelial dysfunction is likely be responsible of both pulmonary in situ platelet aggregation and deep thrombosis potentially leading to severe pulmonary embolism and right ventricular failure(Tavazzi et al., 2020). Coronavirus disease 2019 caused by severe acute respiratory syndrome coronavirus 2 (SARS‐CoV‐2) primarily affects the lungs but can involve any organ. Patients who have recovered from COVID‐19 have presented with complications such as thrombotic episodes in various organs both during and after being infected with SARS‐CoV‐2. A COVID‐19‐associated prothrombotic state has been mentioned in multiple recent research articles (Iqbal et al., 2021). The COVID‐19, due to SARS‐CoV‐2, has uncovered many real‐world issues when it comes to healthcare management and has led to widespread mortality. These include myopericarditis, acute coronary syndromes, thrombosis, arrhythmias, hypertension, and heart failure (Veldtman et al., 2020). Novel coronavirus disease (COVID‐19) has led to a major public health crisis globally. Currently, myocardial damage is speculated to be associated with COVID‐19, which can be seen as one of the main causes of death of patients with COVID‐19 (Liaqat et al., 2021). These cases suggest a possible connection between acute elevated right ventricular afterload and acute respiratory distress in patients affected by SARS‐CoV‐2 (Martínez‐Mateo et al., 2020). Acute coronary syndrome‐ST‐elevation myocardial infarction is a relevant complication of COVID‐19. Due to high levels of pro‐inflammatory mediators, diffuse coronary thrombosis could occur even in patients without cardiac history or comorbidities (Tedeschi et al., 2020).
